# Pre-diagnostic biomarkers and risk of stress-related disorders: a cohort study based on electronic health records

**DOI:** 10.1186/s12916-026-04916-7

**Published:** 2026-06-01

**Authors:** Anna A. Peltola, Esha Khan, Anna Tirkkonen, Mikaela B. von Bonsdorff, Juulia Jylhävä, Jake Lin

**Affiliations:** 1 Faculty of Medicine and Health Technology, Tampere University, Tampere, Finland; 2 Faculty of Sport and Health Sciences, University of Jyväskylä, Jyväskylä, Finland; 3 Gerontology Research Center, University of Jyväskylä, Jyväskylä, Finland; 4 Public Health Research Program, Folkhälsan Research Center, Helsinki, Finland; 5 Department of Medical Epidemiology and Biostatistics , Karolinska Institutet, Stockholm, Sweden; 6 Research Unit of Population Health, Faculty of Medicine, University of Oulu, Oulu, Finland; 7 Faculty of Medicine , University of Helsinki, Helsinki, Finland

**Keywords:** stress-related disorders, biomarkers, hemoglobin, potassium, low-density lipoprotein cholesterol, electronic health records, Cox proportional hazards models

## Abstract

**Background:**

Stress-related disorders are associated with future somatic health conditions, suggesting these disorders involve systemic mechanisms. Yet, evidence from biomarkers remains fragmented across physiological systems. We investigated whether routinely collected laboratory biomarkers are associated with stress-related disorder risk and analyzed their temporal trends before diagnosis.

**Methods:**

We conducted a retrospective cohort study using electronic health records from Central Finland, collected between 2010 and 2023. Our analytical sample included 73,909 individuals: 6,758 cases and 67,151 controls matched on sex and birth year. Cases were diagnosed with stress-related disorders: acute stress reaction, posttraumatic stress disorder, adjustment disorder, other/unspecified reactions to severe stress, burnout, or stress not elsewhere classified. At baseline, participants were aged 34–92 years and were followed for an average of 4.6 ± 3.3 years. Ten routine biomarkers were examined: C-reactive protein, hemoglobin, glucose, glycated hemoglobin, triglycerides, high-density lipoprotein cholesterol (HDL-C), low-density lipoprotein cholesterol (LDL-C), creatinine, sodium, and potassium. Temporal trends were visualized using generalized additive models. Cox proportional hazards models, adjusted for comorbidities, prescribed medications, and care visit frequency, were used to estimate associations between the risk of stress-related disorder diagnosis and biomarker levels measured within one year prior.

**Results:**

Three biomarkers were associated with the risk of stress-related disorder diagnosis in the multivariable model. Higher hemoglobin (HR 0.98 per g/L, 95% CI, 0.97–0.99) and higher potassium (HR 0.74 per mmol/L, 95% CI, 0.64–0.86) were associated with a reduced risk, while higher LDL-C was associated with an increased risk (HR 1.12 per mmol/L, 95% CI, 1.06–1.18). The multivariable model concordance was 0.67. In time-varying models extended over the full follow-up, only hemoglobin retained a significant association.

**Conclusions:**

Hemoglobin, potassium, and LDL-C showed modest but robust associations with stress-related disorder diagnosis. These findings point to physiological domains for future research into the somatic aspects of stress-related disorders.

## Background

Stress-related disorders arise in response to stressful or traumatic events [1]. While defined by psychological criteria, these disorders are predictive of subsequent somatic health conditions, including cardiovascular, immune, metabolic, and kidney disease [2–5]. These associations suggest that stress-related disorders may involve systemic mechanisms, but evidence from biomarkers remains fragmented across physiological systems.

The physiological stress response is primarily regulated by two neuroendocrine systems: the sympathetic nervous system (SNS) and the hypothalamic-pituitary-adrenal (HPA) axis [6]. These systems coordinate cardiovascular, immune, and metabolic functions through the release of catecholamines and glucocorticoids [6–8]. According to the allostatic load model, sustained activation of the SNS and the HPA axis may impair interconnected physiological systems, increasing vulnerability to both psychiatric and somatic health conditions [9–11].

Despite this theoretical understanding, empirical research on stress-related disorders remains limited and uneven across physiological systems. This is particularly true for biomarkers – indicators of biological processes, used to assess disease states and predict progression [12]. Beyond the primary neuroendocrine systems [13, 14], evidence is strongest for immune biomarkers [15, 16], promising but less established for metabolic markers [17, 18], and sparse for other organ systems, such as the renal system [5].

With few exceptions, research has rarely addressed pre-diagnostic biomarkers – those that might reflect early physiological changes prior to disorder onset. One electronic health record study found associations between metabolic markers and stress-related disorders years before diagnosis [17]. More broadly, a prospective cohort study reported associations between a composite biomarker index and other psychiatric outcomes: depression, anxiety, and suicide [19]. Whether individual routine biomarkers across different physiological systems precede stress-related disorders remains unexplored.

Using electronic health records from Central Finland Wellbeing Services County, this study investigates whether routinely collected laboratory biomarkers are associated with future stress-related disorder diagnosis. We focus on biomarkers across five categories: immune (C-reactive protein), hematologic (hemoglobin), metabolic (glucose, lipids), renal (creatinine), and electrolyte (sodium, potassium). These biomarkers were selected for their established roles in general health screening and their frequent availability in clinical practice. The selection reflects the broad state of somatic health, allowing for varied connections to stress physiology.

To identify associations between biomarker levels and subsequent diagnosis, we applied a two-step approach. First, we visualized long-term biomarker trends using generalized additive models (GAMs). Second, we estimated associations between recent biomarker measurements and future diagnoses using Cox proportional hazard models. This allowed us to examine whether common clinical biomarkers reflect measurable physiological changes both transiently and years before stress-related disorder diagnosis.

## Methods

### Study population

The Central Finland Wellbeing Services County provides primary, secondary, and tertiary public healthcare for all residents in the region. Our study population comprises all individuals aged 34 and above whose healthcare contact resulted in at least one laboratory measurement between January 1, 2010, and December 30, 2023 (n = 148,438; 52% female). During this period, 6,758 individuals (4.6%) received a diagnosis of a stress-related disorder. Diagnoses and comorbidities were derived from the Finnish version of the *International Classification of Diseases, Tenth Revision* (ICD-10) [1], and prescribed medications from the *Anatomical Therapeutic Chemical Classification System* (ATC) [20].

### Study design and analytical sample

We conducted a retrospective cohort study with frequency-matched control sampling. From the underlying cohort of 148,438 individuals, all 6,758 cases diagnosed with a stress-related disorder were included. Controls (n = 67,151) were sampled from the remaining individuals to approximate the joint sex and birth year distribution of cases. This resulted in an analytical sample of 73,909 individuals (9.1% cases). Frequency matching was chosen over individual matching to maximize use of available controls while retaining most of the precision gains of exact matching [21]. To maintain comparable follow-up periods between cases and controls, each control was assigned an index date randomly drawn from case diagnosis dates within the same sex-birth-year-stratum. At baseline, participants were aged 34 to 92 years and were followed for an average of 4.6 ± 3.3 years.

### Stress-related disorders

We defined stress-related disorders to include acute stress reaction (ICD-10 code F43.0), posttraumatic stress disorder (PTSD; F43.1), adjustment disorders (F43.2), other reactions to severe stress (F43.8), and unspecified reaction to severe stress (F43.9, F43). We also incorporated the diagnoses of burnout (Z73.0) and stress not elsewhere classified (Z73.3).

### Biomarkers

The biomarkers analyzed included C-reactive protein (CRP), hemoglobin (Hb), fasting glucose, glycated hemoglobin (HbA1c), triglycerides (TG), high-density-lipoprotein cholesterol (HDL-C), low-density-lipoprotein cholesterol (LDL-C), creatinine (Cr), sodium (Na), and potassium (K), all measured from serum samples. Measurements were obtained through clinical care contact – including planned and unplanned care visits – rather than systematic population-level screening.

All laboratory analyses were conducted according to standardized protocols of Central Finland Wellbeing Services County. Before statistical analysis, CRP values > 10 mg/L were excluded to minimize the influence of acute inflammation or infection, as these levels are established markers of acute-phase response rather than chronic systemic inflammation [22]. Potassium values > 10 mmol/L (n = 4) were removed as biologically implausible outliers.

### Covariates

Sex and birth year were included as covariates to adjust for residual imbalance after frequency matching [21]. Chronic somatic comorbidity was quantified using the Charlson Comorbidity Index (CCI) [23], based on 19 weighted ICD-10 codes recorded between the baseline measurement and the index date [24]. Care visit frequency was included as a proxy for informed presence bias – the tendency for individuals with more healthcare contact to have a higher probability of both laboratory testing and diagnosis [25].

Prescribed medications were identified using ATC codes: antidiabetics (A10), lipid-lowering agents (C10), agents acting on the renin-angiotensin system (C09), thiazide diuretics (C03A), loop diuretics (C03C), potassium-sparing diuretics (C03D), antidepressants (N06A), and beta-blockers (C07). These were selected for their potential to influence biomarkers under study and grouped by expected direction and magnitude of effect.

### Analytical strategy

**Fig. 1:**
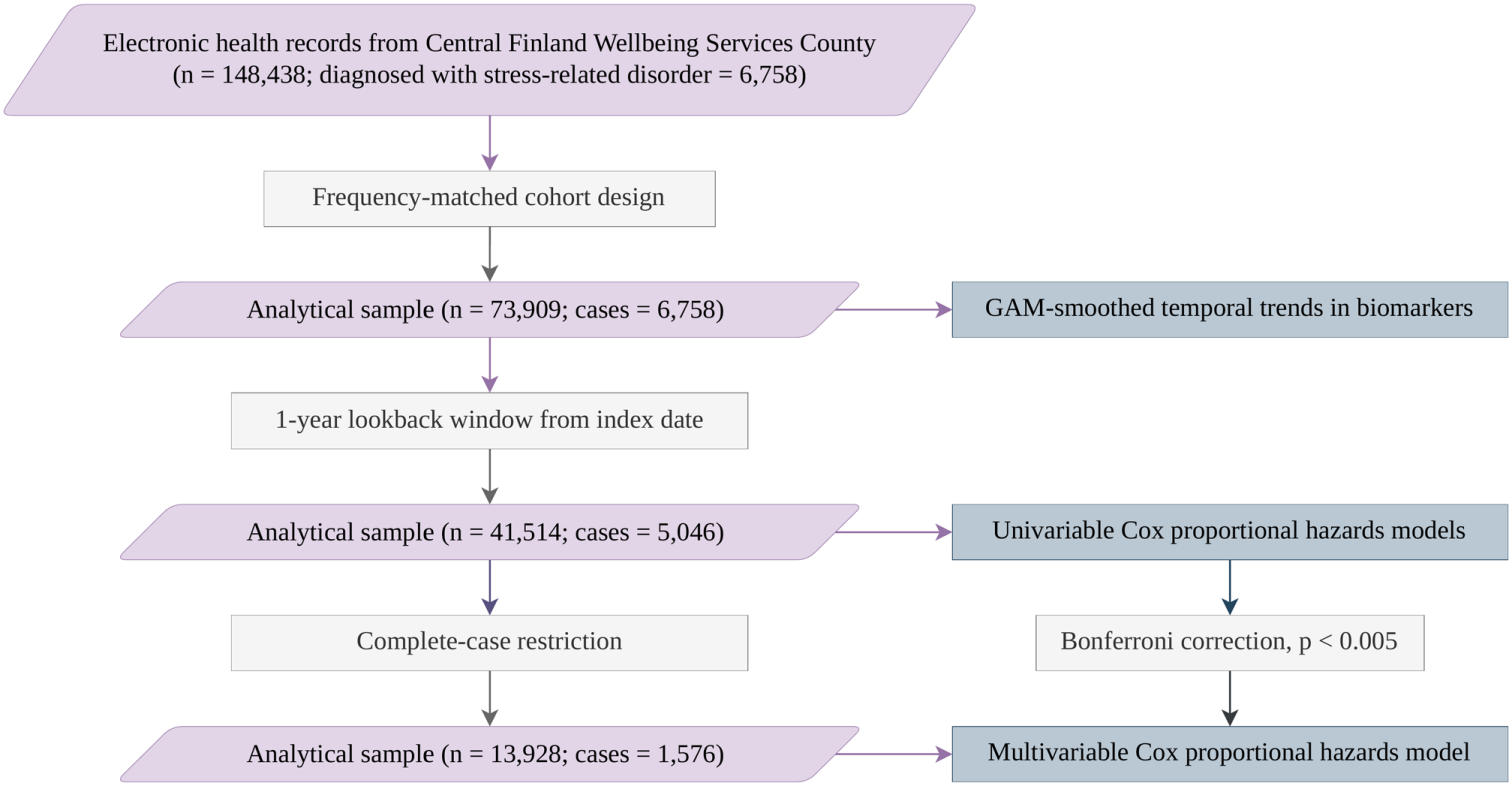
Study workflow describing participant selection and analytical strategy. Abbreviations: GAM, generalized additive model

Our analysis proceeded in two phases. First, we visualized temporal trends in biomarkers before diagnosis using generalized additive models. Second, we estimated associations between biomarker levels and stress-related disorder diagnosis within a 1-year lookback window using Cox proportional hazards models. We then conducted sensitivity analyses to assess the robustness of these associations, described below. The study workflow, including analytical samples at each stage of analysis, is presented in Fig. 1.

#### Descriptive temporal trends

We used generalized additive model smooths (GAMs) to visualize long-term population-level trends in biomarker levels before the index date by case status in the full analytical sample (n = 73,909, cases = 6,758). Smooth terms were specified as cubic regression splines (*k* = 10), and smoothing parameters were selected using generalized cross-validation (GCV). Given the sparse nature of the repeated measures, models were fitted to pooled observations to visualize population-level trends rather than individual-level change. Robustness of these visualizations was assessed through sensitivity analyses, restricting the cohort to individuals with at least two measurements per biomarker.

#### Cox proportional hazards models

GAM visualizations suggested nonlinear biomarker trends and increased divergence between cases and controls as diagnosis approached. Based on these patterns, we selected a 1-year lookback window for the primary analysis, using the most recent biomarker measurement within this window. From the full analytical sample, 41,514 individuals (cases = 5,046) had at least one measurement within this window and were included in subsequent analyses.

We fitted Cox proportional hazards models to estimate associations between biomarker levels and the probability of a stress-related disorder diagnosis. Because the study design used frequency matching rather than individual matching [21], we used unconditional models. Time-to-event was measured from baseline age to age at diagnosis for cases and age at index date for controls. Sex and birth year were included as covariates to adjust for residual imbalance after frequency matching.

We first fitted univariable models for each biomarker. Biomarkers that met the Bonferroni-corrected significance (p < 0.05 divided by 10 tests) were included in the multivariable model. This threshold was applied to reduce the risk of including associations driven by sample size, while remaining achievable for genuine effects given the small number of pre-specified biomarkers and low multicollinearity (variance inflation factors [VIFs]: 1.03–1.34).

The multivariable model additionally adjusted for CCI, care visit frequency (number of visits within the lookback period), and prescribed medications (prescriptions within 1 year before the first laboratory measurement considered for each individual). The model was restricted to complete cases for all exposure variables (n = 13,928; cases = 1,576). Complete-case analysis was considered appropriate because the sample size remained large, baseline biomarker values and comorbidity levels were comparable between included and excluded individuals (Additional file 1, Table S1), and potential sources of selection bias were accounted for as covariates.

Model assumptions were assessed using Schoenfeld residual plots for proportional hazards and VIFs for multicollinearity. Concordance indices with internal train-test 80:20 cross-validation were used to evaluate the predictive performance for both univariable and multivariable models. As a sensitivity analysis, analogous logistic regression models were evaluated using the area under the receiver operating characteristic curve (AUC) with equivalent cross-validation.

#### Sensitivity and subtype analyses

We conducted several sensitivity analyses on the primary multivariable model with the same covariates. To reduce the possibility that observed associations reflect pre-existing conditions, we applied a 1-year washout period, excluding cases with less than one year between their first measurement and diagnosis. To assess robustness to diagnostic heterogeneity, we first excluded individuals with Z73 codes (n = 113), which may capture milder stress complaints, and then additionally excluded individuals with PTSD (n = 105), which differs from other stress-related disorders in its chronicity and specific trauma-exposure criteria. We further performed exploratory subtype-specific analyses for the three largest diagnostic subgroups: acute stress reaction (n = 580), adjustment disorder (n = 538), and a combined group of other and unspecified reactions to severe stress (n = 241).

Separately, to examine whether associations persisted over longer follow-up, we fitted univariable time-varying Cox models to the Cox-eligible sample (n = 41,514) using all available measurements over the full follow-up period.

#### Infrastructure

All data were stored and analyzed within the secure server environment of the Helsinki-Uusimaa Hospital District (HUS). Analyses were performed using R version 4.5.0, with packages including *ggplot2 3.5.2*, *gridExtra 2.3, caret 7.0.1*, *survival 3.8.3*, and *rms 8.0.0*.

## Results

### Descriptive results

The analytical sample included 73,909 participants, of whom 6,758 (9.1%) were cases. Cases were younger than controls at baseline (53.3 ± 12.2 vs. 57.8 ± 12.8 years) and more frequently female (73.6% vs. 64.4%). Comorbidity levels were low and comparable between groups. Baseline biomarker levels were similar across cases and controls (Table 1; for sample sizes by biomarker, see Additional file 1, Table S2). Among cases, the most common first recorded diagnoses were acute stress reaction (38.6%) and adjustment disorders (33.5%) (Additional file 1, Table S3).

**Table 1:** Baseline sample characteristics by case status

	All	Cases	Controls
**Demographics**			
Age (mean ± SD)	57.4 ± 12.8	53.3 ± 12.2	57.8 ± 12.8
Female, n (%)	48,207 (65.2%)	4,975 (73.6%)	43,232 (64.4%)
Male, n (%)	25,702 (34.8%)	1,783 (26.4%)	23,919 (35.6%)
**Follow-up**			
Follow-up, years (mean ± SD)	4.6 ± 3.3	4.2 ± 3.4	4.6 ± 3.3
**Comorbidity**			
CCI (median, IQR)^a^	0.00 (0.00–1.00)	0.00 (0.00–1.00)	0.00 (0.00–1.00)
**Biomarkers**			
CRP, mg/L (median, IQR)	2.00 (1.00–4.00)	2.00 (1.00–4.00)	2.00 (1.00–4.00)
Hb, g/L (median, IQR)	138.00 (130.00–147.00)	137.00 (129.00–146.00)	139.00 (130.00–147.00)
HbA1c, mmol/mol (mean ± SD)	42.47 ± 11.30	41.66 ± 11.39	42.56 ± 11.29
Glucose, mmol/L (median, IQR)	5.70 (5.30–6.20)	5.60 (5.20–6.10)	5.70 (5.30–6.20)
TG, mmol/L (median, IQR)	1.10 (0.80–1.60)	1.10 (0.80–1.60)	1.10 (0.80–1.60)
LDL-C, mmol/L (mean ± SD)	2.98 ± 0.95	3.01 ± 0.92	2.97 ± 0.95
HDL-C, mmol/L (mean ± SD)	1.56 ± 0.47	1.56 ± 0.46	1.56 ± 0.48
Cr, µmol/L (median, IQR)	70.00 (61.00–80.00)	68.00 (59.00–77.00)	70.00 (61.00–81.00)
Sodium, mmol/L (mean ± SD)	140.79 ± 3.19	140.67 ± 2.98	140.81 ± 3.21
K, mmol/L (mean ± SD)	3.97 ± 0.37	3.92 ± 0.35	3.95 ± 0.37

Categorical variables are presented as counts (n) and percentages (%); normally distributed continuous variables as mean ± standard deviation (SD); and non-normally distributed variables as median and interquartile range (IQR). CCI is reported at the index date instead of at baseline. All biomarker values are expressed in SI units. Abbreviations: CCI, Charlson Comorbidity Index; CRP, C-reactive protein; Hb, hemoglobin; HbA1c, glycated hemoglobin; TG, triglycerides; LDL-C, low-density-lipoprotein cholesterol; HDL-C, high-density-lipoprotein cholesterol; Cr, Creatinine; K, Potassium

Of the 41,514 individuals with at least one measurement within the 1-year lookback window, 13,928 had complete data for all exposure variables and were included in the multivariable model. These individuals were older on average but had comparable biomarker values (Additional file 1, Table S1). Within the 1-year lookback window, the most recent measurement before the index date was consistently closer in time for cases than controls across all biomarkers (Additional file 1; Table S4), likely reflecting increased clinical monitoring approaching diagnosis. This difference was largest for CRP, hemoglobin, creatinine, and electrolytes (median 43–54 days for cases vs. 118–137 days for controls), which are among the most commonly ordered laboratory tests in routine care, and smaller for metabolic markers (median 118–134 days for cases vs. 141–153 days for controls), which are typically ordered in planned primary care visits.

### Temporal trends in biomarkers

**Fig. 2:**
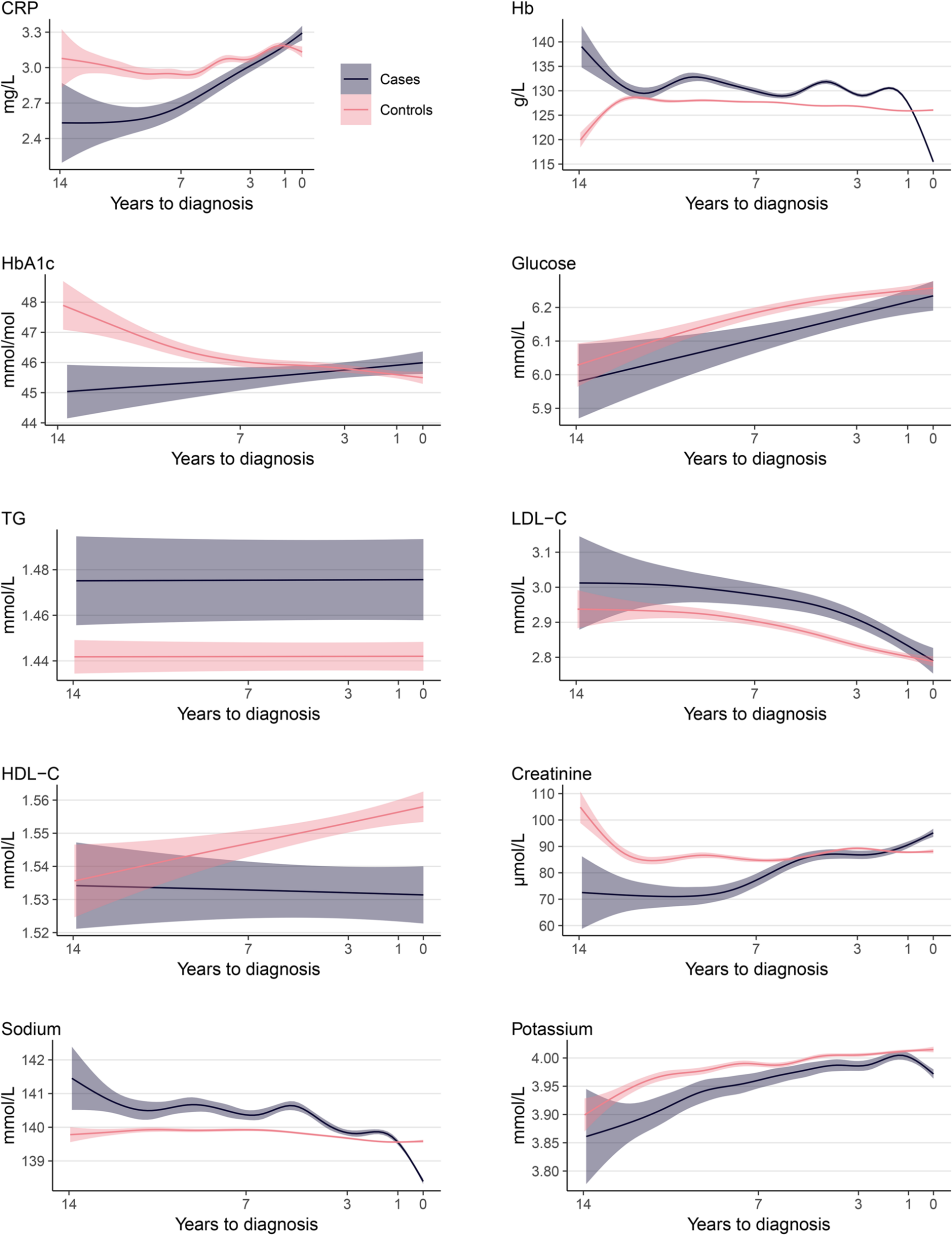
Population-level temporal trends of 10 biomarkers by case status. The horizontal axis represents years before the diagnosis (assigned index date for controls). Trends were estimated using generalized additive models (GAMs) with cubic regression splines (*k* = 10) fitted to pooled observations. Cases are shown in dark blue and controls in pink; shaded areas indicate 95% confidence intervals. Each biomarker is plotted against its own range to maintain visual comparability across different units. Measurement frequency and timing vary by biomarker. Abbreviations: CRP, C-reactive protein; Hb, hemoglobin; HbA1c, glycated hemoglobin; TG, triglycerides; LDL-C, low-density-lipoprotein cholesterol; HDL-C, high-density-lipoprotein cholesterol

GAM charts captured population-level temporal trends in biomarker levels throughout follow-up (Fig. 2). In the months approaching diagnosis, hemoglobin, potassium, and sodium showed declines in mean values for cases. Lipid markers showed minor deviations between group means over a longer timeframe. These patterns were consistent when restricting the analysis to individuals with at least two measurements (Additional file 1, Fig. S1) and they informed the selection of a 1-year lookback window for the subsequent Cox proportional hazards models.

### Cox proportional hazards models

In univariable models adjusted for sex and birth year, six of ten biomarkers reached the Bonferroni-corrected significance threshold and were included in the multivariable model: hemoglobin (HR 0.98 per g/L, 95% CI 0.98–0.98), glucose (HR 0.94 per mmol/L, 95% CI 0.91–0.97), LDL-C (HR 1.10 per mmol/L, 95% CI 1.06–1.15), HDL-C (HR 0.86 per mmol/L, 95% CI 0.78–0.94), sodium (HR 0.98 per mmol/L, 95% CI 0.98–0.99), and potassium (HR 0.68 per mmol/L, 95% CI 0.63–0.75) (Table 2). Cross-validated concordance indices for these individual biomarkers ranged from 0.57 to 0.62, and analogous logistic regression models produced AUCs in a similar range (Additional file 1, Table S5).

**Table 2:** Results from 10 univariable Cox proportional hazards models (Y = stress-related disorder diagnosis), each adjusted for sex and birth year

	HR per unit	95% CI	HR per IQR	95% CI	p	No. of cases
**Exposure**						
CRP	0.99	(0.97–0.99)	0.97	(0.92–1.01)	0.113	2,816
**Hb**	**0.98**	**(0.98–0.98)**	**0.72**	**(0.69–0.74)**	**<0.001**	**4,892**
HbA1c	1.00	(1.00–1.00)	1.03	(0.99–1.06)	0.156	1,955
**Glucose**	**0.94**	**(0.91–0.97)**	**0.94**	**(0.91–0.97)**	**<0.001**	**2,279**
TG	0.93	(0.89–0.99)	0.96	(0.92–0.99)	0.012	2,321
**LDL-C**	**1.10**	**(1.06–1.15)**	**1.14**	**(1.07–1.20)**	**<0.001**	**2,290**
**HDL-C**	**0.86**	**(0.78–0.94)**	**0.90**	**(0.84–0.96)**	**0.001**	**2,354**
Creatinine	1.00	(1.00–1.00)	0.97	(0.95–0.98)	0.010	4,514
**Sodium**	**0.98**	**(0.98–0.99)**	**0.94**	**(0.91–0.98)**	**0.002**	**4,176**
**Potassium**	**0.68**	**(0.63–0.75)**	**0.86**	**(0.83–0.89)**	**<0.001**	**4,186**

Exposure variables assessed in 1-year lookback window prior to diagnosis. HR per IQR scales each biomarker to its interquartile range for cross-biomarker comparison. Abbreviations: HR, hazard ratio; CI, confidence interval; IQR, interquartile range; CRP, C-reactive protein; Hb, hemoglobin; HbA1c, glycated hemoglobin; TG, triglycerides; LDL-C, low-density-lipoprotein cholesterol; HDL-C, high-density-lipoprotein cholesterol

In the multivariable model, additionally adjusted for CCI, care visit frequency, and prescribed medications, three biomarkers remained significant (Table 3). Higher hemoglobin (HR 0.98 per g/L, 95% CI 0.97–0.99) and higher potassium (HR 0.74 per mmol/L, 95% CI 0.64–0.86) were associated with reduced risk, while higher LDL-C was associated with increased risk (HR 1.12 per mmol/L, 95% CI 1.06–1.18). When scaled to interquartile ranges, hemoglobin showed the strongest effect (HR 0.71), followed by LDL-C (HR 1.16) and potassium (HR 0.89). The concordance index for the multivariable model was 0.67 (cross-validated: 0.67), and analogous logistic regression produced AUCs of 0.72–0.74 (Additional file 1, Table S5). The proportional hazards assumption was supported by Schoenfeld residual plots and multicollinearity was low (VIFs 1.03–1.34; Additional file 1, Table S6).

**Table 3:** Results from multivariable Cox proportional hazards model (Y = stress-related disorder diagnosis), adjusted for Charlson comorbidity index, medications, care visit frequency, sex, and birth year (n = 13,928; cases = 1,576)

	HR per unit	95% CI	HR per IQR	95% CI	p
**Exposure**					
**Hb**	**0.98**	**(0.97–0.99)**	**0.71**	**(0.66–0.76)**	**<0.001**
Glucose	0.99	(0.95–1.03)	0.99	(0.96–1.03)	0.686
**LDL-C**	**1.12**	**(1.06–1.18)**	**1.16**	**(1.08–1.24)**	**<0.001**
HDL-C	0.91	(0.81–1.02)	0.94	(0.86–1.01)	0.130
Sodium	0.99	(0.98–1.01)	0.98	(0.91–1.05)	0.552
**Potassium**	**0.74**	**(0.64–0.86)**	**0.89**	**(0.83–0.94)**	**<0.001**
**Model fit**					
*Concordance*	0.674				

Exposure variables assessed in a 1-year lookback window, restricted to complete cases. HR per IQR scales each biomarker to its interquartile range for cross-biomarker comparison. Abbreviations: HR, hazard ratio; CI, confidence interval; IQR, interquartile range; Hb, hemoglobin; LDL-C, low-density-lipoprotein cholesterol; HDL-C, high-density-lipoprotein cholesterol

### Sensitivity analyses

Results in the multivariable model remained essentially unchanged when applying a 1-year washout period (Additional file 1, Table S7), when excluding Z73 diagnoses (Additional file 1, Table S8), and when additionally excluding PTSD diagnoses (Additional file 1, Table S8). In subtype-specific analyses for acute stress reaction, adjustment disorder, and other and unspecified reactions to severe stress, the direction of effects was consistent, though some associations attenuated to non-significance in the smaller subgroups (Additional file 1, Table S9).

In supplementary univariable time-varying Cox models extended over the full follow-up, only hemoglobin retained a significant association (HR 0.99 per g/L, 95% CI 0.99–0.99), while potassium and LDL-C attenuated to null (Additional file 1, Table S10).

## Discussion

Three biomarkers were associated with stress-related disorder diagnosis within one year before onset, and these associations remained robust to adjustment for prescribed medications and somatic comorbidities. Higher potassium was associated with a reduced risk of stress-related disorders (HR 0.74 per mmol/L, 95% CI 0.64–0.86), as was higher hemoglobin (HR 0.98 per g/L, 95% CI 0.97–0.99). Higher LDL-C was associated with an increased risk (HR 1.12 per mmol/L, 95% CI 1.06–1.18). When scaled to interquartile ranges for cross-biomarker comparison, hemoglobin showed the strongest effect (HR 0.71), followed by LDL-C (HR 1.16) and potassium (HR 0.89).

The concordance index for the primary multivariable model was 0.67, where 0.50 indicates no discrimination and values above 0.70 are generally considered clinically actionable [26]. Stress-related disorders develop in response to external events that cannot be anticipated from electronic health records, placing a ceiling on prediction from biomarkers and clinical covariates alone. While these metrics do not support standalone clinical prediction, they suggest that routine biomarkers are associated with stress-related disorder diagnosis. Below, we contextualize each significant biomarker against the closest available evidence, noting that mechanistic interpretations are offered as plausible explanations given the observational design.

To our knowledge, our study is the first to have examined potassium levels in relation to stress-related disorders. Higher potassium levels showed a modest protective association, and temporal trend analysis suggested potassium levels declined close to diagnosis. Two potential pathways could explain these observations. First, acute catecholamine elevations, present in the stress response, can transiently shift potassium from bloodstream into cells, lowering serum levels [27, 28]. Second, and more plausibly for sustained effects, catecholamines also stimulate renin release [29], while HPA axis activity stimulates aldosterone secretion [30, 31]. Through the renin-angiotensin-aldosterone system (RAAS), aldosterone induces renal potassium excretion [32]. The RAAS pathway provides a plausible framework for our potassium findings, as its dysregulation contributes to the same conditions predicted by stress-related disorders – cardiovascular, metabolic, and kidney disease [2, 4, 5, 32]. RAAS activity has also been associated with trauma exposure and PTSD [33]. However, this interpretation remains one plausible framework among several, as potassium levels are influenced by various factors, such as diet and renal function.

Hemoglobin has, to our knowledge, not been studied in relation to stress-related disorders. In our study, it showed the strongest relative effect (HR 0.71 per IQR) and was the only significant biomarker to retain an association in time-varying Cox models extended over the full follow-up. Hemoglobin may serve as a broad indicator of overall health status, as it is influenced by multiple physiological factors, including inflammation, iron metabolism, nutrition, and kidney function. The absence of an association with CRP challenges a straightforward inflammatory explanation. However, CRP has been less consistently associated with stress-related disorders than pro-inflammatory cytokines [15], and immune-related processes not captured by CRP – including altered iron metabolism – cannot be excluded from interpretation [34].

LDL-C has been examined in previous studies with varied results. The only previous longitudinal study found no significant association for LDL-C years before diagnosis [17], whereas a meta-analysis of predominantly cross-sectional studies reported an association between LDL-C and PTSD [18]. Our finding of increased risk within the 1-year lookback aligns with the cross-sectional evidence but attenuated to null in time-varying models, suggesting a short-term signal. This temporal pattern could be explained by glucocorticoid-mediated effects on lipid metabolism [31], which would be expected closer to disease onset. Given the established role of LDL-C in, e.g., cardiovascular and metabolic disease – conditions that stress-related disorders are associated with [2, 4] – this association merits further research.

Taken together, these findings suggest that three routine biomarkers from different physiological systems show independent associations with stress-related disorder diagnosis. While the observational design precludes causal inference, the findings may inform future causally oriented research into the somatic dimensions of stress-related pathology.

### Limitations

Several limitations should be considered in the interpretation of the findings. Our data are limited to clinical variables recorded routinely in electronic health records within a single Finnish region. For this reason, we could not account for lifestyle and social factors – e.g., diet, body composition, smoking, alcohol use, and socioeconomic position – that may influence both biomarker levels and vulnerability to stress-related disorders. Although we adjusted for comorbidities and prescribed medications that may directly affect the biomarkers under study, residual confounding from unmeasured variables remains a possibility.

Stressful and traumatic events, central to the etiology of stress-related disorders, are not captured in structured health records. The pathway from a stressful event to symptom onset to recorded diagnosis may vary by individual, and none of these transitions are directly observable in our data. The biomarker associations within the 1-year lookback window may therefore reflect pre-existing physiological vulnerability, an ongoing but undiagnosed symptomatic period, or both – though in either case, the associations precede recorded diagnosis. The attenuation of most associations in time-varying models, accounting for longer-term associations, suggests that these associations appear mostly close to diagnosis, but our study cannot distinguish between these explanations.

As with EHR research in general, our sample is conditioned on healthcare contact and having sufficient observations, which may overrepresent individuals with poorer health or higher healthcare utilization [25, 35]. We included comorbidity and care-visit frequency as covariates to mitigate informed presence bias, and although associations were robust to these adjustments, residual bias may remain. Controls are also not confirmed free of stress-related symptoms, as individuals with subclinical or undiagnosed conditions would remain in the control group, which may dilute observed differences between groups. Additionally, although public healthcare is tax-funded and accessible to all individuals in Finland, those who rely primarily on private or occupational care – who are more likely to be higher-income and employed – may be slightly underrepresented in our study population [36, 37].

Finally, model discrimination was modest and evaluation was limited to a single cohort without external validation. These findings are therefore best viewed as hypothesis-generating rather than clinically actionable.

## Conclusions

In this large EHR-based cohort, hemoglobin, potassium, and LDL-C showed modest but robust associations with stress-related disorder risk in the year preceding diagnosis. These exploratory findings suggest physiological domains for future research into the somatic aspects of stress-related disorders.

## Supplementary information

Additional file 1 Supplementary material, Tables S1–S10 and Fig. S1. Table S1 – Baseline characteristics by inclusion in Cox proportional hazards models. Table S2 – Sample sizes by biomarker. Table S3 – Distribution of stress-related disorder diagnoses. Table S4 – Time from most recent biomarker measurement to index date. Fig. S1 – Sensitivity analysis of GAM smooths. Table S5 – Cross-validation and model performance metrics. Table S6 – Variance inflation factors for the multivariable model. Table S7 – Sensitivity analysis applying 1-year washout period. Table S8 – Sensitivity analysis for diagnostic heterogeneity. Table S9 – Subtype-specific analyses. Table S10 – Time-varying Cox models.

Additional file 2 STROBE statement.

## Data Availability

The electronic healthcare records analyzed in this study are not publicly available because they contain sensitive health-related information. Access to these data requires approval from the Central Finland Wellbeing County.

## Abbreviations

ATC: Anatomical Therapeutic Chemical Classification System
AUC: area under the receiver operating characteristic curve
CCI: Charlson Comorbidity Index
CI: confidence interval
Cr: creatinine
CRP: C-reactive protein
EHR: electronic health record
GAM: generalized additive model
GCV: generalized cross-validation
Hb: hemoglobin
HbA1c: glycated hemoglobin
HDL-C: high-density lipoprotein cholesterol
HPA: hypothalamic-pituitary-adrenal
HR: hazard ratio
ICD-10: International Classification of Diseases, Tenth Revision
IQR: interquartile range
K: potassium
LDL-C: low-density lipoprotein cholesterol
Na: sodium
PTSD: posttraumatic stress disorder
RAAS: renin-angiotensin-aldosterone system
SD: standard deviation
SNS: sympathetic nervous system
TG: triglycerides
VIF: variance inflation factor
