# Supplementary material for: Pre-diagnostic biomarkers and risk of stress-related disorders: a cohort study based on electronic health records
Source: BMC Med. 2026 Jun 1;24:337. doi: 10.1186/s12916-026-04916-7 (PMC13227757; doi:10.1186/s12916-026-04916-7)
Supplement: Supplementary file 1 — Additional file 1 Supplementary material, Tables S1–S10 and Fig. S1. Table S1 – Baseline characteristics by inclusion in Cox proportional hazards models. Table S2 – Sample sizes by biomarker. Table S3 – Distribution of stress-related disorder diagnoses. Table S4 – Time from most recent biomarker measurement to index date. Fig. S1 – Sensitivity analysis of GAM smooths. Table S5 – Cross-validation and model performance metrics. Table S6 – Variance inflation factors for the multivariable model. Table S7 – Sensitivity analysis applying 1-year washout period. Table S8 – Sensitivity analysis for diagnostic heterogeneity. Table S9 – Subtype-specific analyses. Table S10 – Time-varying Cox models. [file 12916_2026_4916_MOESM1_ESM.pdf]

Supplementary material

Contents

Descriptive results 1
Table S1. Baseline characteristics by inclusion in Cox proportional hazards models 3
Table S2. Sample sizes by biomarker 1
Table S3. Distribution of stress-related disorder diagnoses 2
Table S4. Time from most recent biomarker measurement to index date 4
Fig. S1. Sensitivity analysis of GAM smooths 5

Cox proportional hazards models 6
Table S5. Cross-validation and model performance metrics 6
Table S6. Variance inflation factors for the multivariable model 7
Table S7. Sensitivity analysis applying 1-year washout period 8
Table S8. Sensitivity analysis for diagnostic heterogeneity 9
Table S9. Subtype-specific analyses 10
Table S10. Time-varying Cox models 11

Table S1. Baseline characteristics of the individuals with at least one measurement within the 1-year lookback window (n = 41,514), presented by case status and by complete versus incomplete data for the multivariable model.

	Complete case (n = 13,928)		Incomplete case (n = 27,586)	
	Case	Control	Case	Control
**Demographics**				
Age, mean ± SD	57.1 ± 12.0	62.0 ± 11.2	54.1 ± 12.8	59.5 ± 13.1
Female, n (%)	1,106 (70.2%)	8,387 (67.9%).	2,531 (73.0%)	15,864 (65.8%)
Male, n (%)	470 (29.8%)	3,965 (32.1%)	939 (27.0%)	8,252 (34.2%)
**Follow-up**				
Follow-up, years (mean ± SD)	4.4 ± 3.5	4.4 ± 3.3	3.7 ± 3.4	3.8 ± 3.3
**Comorbidity**				
CCI (median, (IQR)	0.00 (0.00–1.00)	0.00 (0.00–1.00)	0.00 (0.00–1.00)	0.00 (0.00–1.00)
**Biomarkers**				
CRP, mg/L (median, IQR)	2.00 (1.00–4.00)	2.00 (1.00–4.00)	2.00 (1.00–4.00)	2.00 (1.00–4.00)
Hb, g/L (median, IQR)	138 (130–147)	140 (132–148)	137 (128–145)	138 (129–146)
HbA1c, mmol/mol (mean ± SD)	41.99 ± 10.54	42.39 ± 9.72	42.62 ± 12.59	43.48 ± 12.36
Glucose, mmol/L (median, IQR)	5.70 (5.30–6.30)	5.70 (5.30–6.30)	5.60 (5.20–6.10)	5.70 (5.30–6.20)
TG, mmol/L (median, IQR)	1.20 (0.90–1.70)	1.20 (0.90–1.60)	1.10 (0.80–1.60)	1.10 (0.80.160)
LDL-C, mmol/L (mean ± SD)	2.98 ± 0.94	2.94 ± 0.96	2.99 ± 0.93	2.94 ± 0.96
HDL-C, mmol/L (mean ± SD)	1.54 ± 0.46	1.56 ± 0.47	1.56 ± 0.46	1.56 ± 0.48
Cr, µmol/L (median, IQR)	69.00 (60.00–79.00)	70.00 (61.00–81.00)	67.00 (59.00–78.00)	70.00 (61.00–81.00)
Sodium, mmol/L (mean ± SD)	140.86 ± 3.00	141.31 ± 2.76	140.52 ± 3.13	140.70 ± 3.31
K, mmol/L (mean ± SD)	3.94 ± 0.35	3.96 ± 0.34	3.92 ± 0.36	3.96 ± 0.37

Abbreviations: SD, standard deviation; CCI, Charlson Comorbidity Index; IQR, interquartile range; CRP, C-reactive protein; Hb, hemoglobin; HbA1c, glycated hemoglobin; TG, triglycerides; LDL-C, low-density lipoprotein cholesterol; HDL-C, high-density lipoprotein cholesterol.

Table S2. Sample sizes by biomarker and case status in the full analytical sample (n = 73,909).

	All	Cases	Controls
**Demographics**			
Age	73,909	6,758	67,151
Female	48,207	4,975	43,232
Male	25,702	1,783	23,919
**Comorbidity**			
CCI	73,909	6,758	67,151
**Biomarkers**			
CRP	46,744	4,883	41,861
Hb	72,442	6,664	65,778
HbA1c	33,707	3,395	30,312
Glucose	41,454	4,334	37,120
TG	42,869	4,357	38,512
LDL-C	42,420	4,315	38,105
HDL-C	43,170	4,377	38,739
Creatinine	66,897	6,205	60,692
Sodium	63,225	5,960	57,265
Potassium	63,306	5,973	57,333

Abbreviations: CCI, Charlson Comorbidity Index; CRP, C-reactive protein; Hb, hemoglobin; HbA1c, glycated hemoglobin; TG, triglycerides; LDL-C, low-density lipoprotein cholesterol; HDL-C, high-density lipoprotein cholesterol; Cr, creatinine.

Table S3. Distribution of stress-related disorder diagnoses (ICD-10) across analytical samples: full analytical sample (n = 73,909), individuals with at least one measurement within the 1-year lookback window (n = 41,514), and complete-case sample (n = 13,928).

	Full sample	1-year lookback	Complete case
**Stress-related disorder, any**	6,758	5,046	1,576
**Category F43**			
Acute stress reaction (F43.0)	2,611	1,749	580
Posttraumatic stress disorder (F43.1)	375	276	105
Adjustment disorders (F43.2)	2,261	1,914	538
Other reactions to severe stress (F43.8)	408	312	93
Unspecified reaction to severe stress (F43.9, F43)	644	489	147
**Category Z73**			
Burnout (Z73.0)	308	202	68
Stress not elsewhere classified (Z73.3)	151	104	45

Abbreviations: ICD-10, International Classification of Diseases, Tenth Revision.

Table S4. Days (median, IQR) from the most recent laboratory measurement to the index date, by case status and analytical sample.

	Full sample (n = 73,909)		1-year lookback (n = 41,514)	
	Cases	Controls	Cases	Controls
**Biomarkers**				
CRP	242 (34–815)	623 (213–1386)	54 (9–164)	137 (56–239)
Hb	112 (14–382)	331 (109–952)	45 (5–153)	118 (46–221)
HbA1c	274 (94–700)	369 (142–934)	118 (40–219)	141 (65–240)
Glucose	334 (126–791)	411 (166–1019)	134 (48–231)	153 (72–251)
TG	328 (115–770)	403 (163–1007)	126 (44–231)	153 (71–251)
LDL-C	329 (117–777)	404 (164–1007)	129 (45–231)	153 (71–252)
HDL-C	321 (113–761)	397 (161–999)	126 (43–229)	153 (71–251)
Creatinine	115 (14–400)	325 (110–927)	43 (6–149)	120 (48–222)
Sodium	134 (15–447)	364 (129–988)	47 (6–157)	130 (54–232)
Potassium	134 (16–447)	363 (129–987)	47 (6–157)	130 (54–232)

Abbreviations: IQR, interquartile range; CRP, C-reactive protein; Hb, hemoglobin; HbA1c, glycated hemoglobin; TG, triglycerides; LDL-C, low-density lipoprotein cholesterol; HDL-C, high-density lipoprotein cholesterol.

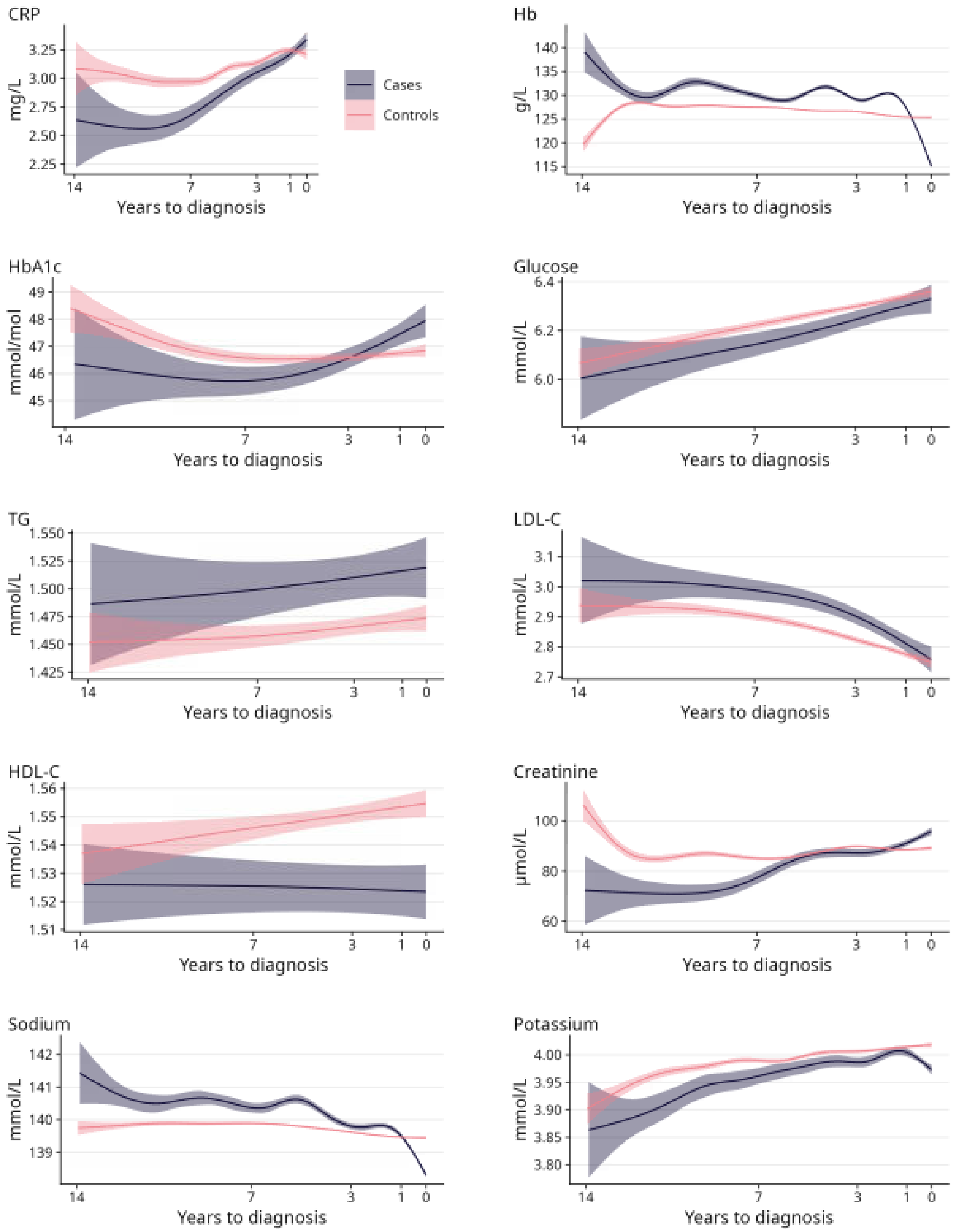

Fig. S1. Sensitivity analysis of GAM smooths, restricted to individuals with at least two measurements per biomarker.

Abbreviations: GAM, generalized additive model; CRP, C-reactive protein; Hb, hemoglobin; HbA1c, glycated hemoglobin; TG, triglycerides; LDL-C, low-density lipoprotein cholesterol; HDL-C, high-density lipoprotein cholesterol.

Table S5. Cross-validated performance metrics (80:20 train-test split) for univariable and multivariable models. Performance is reported as concordance index for Cox proportional hazards models and AUC for analogous logistic regression models.

	Concordance		AUC	
	Train	*Test*	*Train*	*Test*
**Univariable**				
CRP	0.56	0.55	0.60	0.57
Hb	0.62	0.62	0.65	0.65
HbA1c	0.63	0.63	0.60	0.61
Glucose	0.60	0.62	0.63	0.63
TG	0.60	0.59	0.61	0.61
LDL-C	0.60	0.60	0.61	0.62
HDL-C	0.60	0.59	0.61	0.62
Creatinine	0.57	0.57	0.62	0.61
Sodium	0.57	0.58	0.63	0.63
Potassium	0.58	0.59	0.62	0.62
**Multivariable**				
Combined model	0.67	0.67	0.72	0.74

Abbreviations: AUC, area under the receiver operating characteristic curve.

Table S6. Variance inflation factors (VIFs) for all variables included in the multivariable Cox proportional hazards model.

	VIF
**Biomarkers**	
Hb	1.34
Glucose	1.20
LDL-C	1.08
HDL-C	1.15
Sodium	1.12
Potassium	1.03
**Medications**	
Antidiabetics	1.17
Lipid-lowering agents	1.08
Agents acting on RAAS	1.06
Thiazide diuretics	1.01
Loop diuretics	1.06
Potassium-sparing diuretics	1.01
Antidepressants	1.05
Beta-blockers	1.10
**Comorbidity**	
CCI	1.36
**Healthcare utilization**	
Care visit frequency	1.34
**Demographics**	
Sex	1.28
Birth year	1.04

Abbreviations: VIF, variance inflation factor; Hb, hemoglobin; LDL-C, low-density lipoprotein cholesterol; HDL-C, high-density lipoprotein cholesterol; RAAS, renin-angiotensin-aldosterone system; CCI, Charlson Comorbidity Index.

Table S7. Sensitivity analysis with a 1-year washout period for the multivariable Cox proportional hazards model. Cases with less than one year between baseline measurement and diagnosis were excluded (n exluded = 211). The model is adjusted for the same covariates as the primary multivariable model. Analyses were restricted to complete cases for all exposure variables (n = 13,717; no. of cases = 1,365).

	HR per unit	95 % CI	p
Hb	0.98	(0.98–0.99)	<0.001
Glucose	0.97	(0.92–1.01)	0.136
LDL-C	1.12	(1.06–1.20)	<0.001
HDL-C	0.93	(0.82–1.05)	0.255
Sodium	1.00	(0.99–1.02)	0.649
Potassium	0.81	(0.69–0.95)	0.010

Abbreviations: HR, hazard ratio; CI, confidence interval; Hb, hemoglobin; LDL-C, low-density lipoprotein cholesterol; HDL-C, high-density lipoprotein cholesterol.

Table S8. Sensitivity analysis for diagnostic heterogeneity in the multivariable model. Model 1 excludes Z73 codes (n = 13,815; no. of cases = 1,463). Model 2 additionally excludes PTSD (n = 13,370; no. of cases = 1,358). The models are adjusted for the same covariates as the primary multivariable model.

	HR per unit	95 % CI	p
**Model 1**			
Hb	0.98	(0.98–0.98)	<0.001
Glucose	0.99	(0.95–1.03)	0.549
LDL-C	1.11	(1.05–1.17)	<0.001
HDL-C	0.92	(0.81–1.04)	0.164
Sodium	0.99	(0.98–1.01)	0.470
Potassium	0.73	(0.63–0.85)	<0.001
**Model 2**			
Hb	0.98	(0.98–0.98)	<0.001
Glucose	0.99	(0.95–1.04)	0.806
LDL-C	1.11	(1.05–1.18)	<0.001
HDL-C	0.91	(0.80–1.03)	0.122
Sodium	0.99	(0.97–1.01)	0.275
Potassium	0.71	(0.60–0.83)	<0.001

Abbreviations: HR, hazard ratio; CI, confidence interval; Hb, hemoglobin; LDL-C, low-density lipoprotein cholesterol; HDL-C, high-density lipoprotein cholesterol; PTSD, posttraumatic stress disorder.

Table S9. Subtype-specific analyses for the three largest diagnostic subgroups in the multivariable model: acute stress reaction (F43.0; n = 580), adjustment disorder (F43.2; n = 538), and other and unspecified reactions to severe stress (F43.8, F43.9, F43; n = 241). The model is adjusted for the same covariates as the primary multivariable model.

	HR per unit	95 % CI	p
**Acute stress reaction (n = 580)**			
Hb	0.99	(0.98–0.99)	<0.001
Glucose	1.03	(0.97–1.10)	0.376
LDL-C	1.11	(1.01–1.21)	0.032
HDL-C	1.07	(0.88–1.29)	0.519
Sodium	1.03	(0.99–1.06)	0.157
Potassium	0.89	(0.68–1.14)	0.363
**Adjustment disorders (n = 538)**			
Hb	0.97	(0.97–0.98)	<0.001
Glucose	0.99	(0.93–1.05)	0.701
LDL-C	1.10	(1.00–1.21)	0.054
HDL-C	0.78	(0.65–0.98)	0.029
Sodium	0.96	(0.93–0.98)	0.002
Potassium	0.53	(0.42–0.68)	<0.001
**Other and unspecified reactions to severe stress[1] (n = 240)**			
Hb	0.98	(0.97–0.99)	<0.001
Glucose	0.93	(0.83–1.05)	0.229
LDL-C	1.14	(0.99–1.32)	0.061
HDL-C	0.80	(0.59–1.09)	0.171
Sodium	0.99	(0.95–1.04)	0.731
Potassium	0.82	(0.57–1.19)	0.302

Abbreviations: HR, hazard ratio; CI, confidence interval; Hb, hemoglobin; LDL-C, low-density lipoprotein cholesterol; HDL-C, high-density lipoprotein cholesterol.

Table S10. Results from 10 univariable time-varying Cox proportional hazards models, adjusted for sex and birth year, fitted to the sample of individuals with at least one measurement within the 1-year lookback window (n = 41,514) using all available measurements over the full follow-up.

	HR per unit	95 % CI	p	No. of cases
CRP	1.01	(1.00–1.03)	0.080	3,065
Hb	0.99	(0.99–0.99)	<0.001	4,128
HbA1c	1.00	(1.00–1.00)	0.990	2,340
Glucose	1.00	(0.98–1.02)	0.944	2,892
TG	1.00	(0.96–1.04)	0.921	2,917
LDL-C	0.99	(0.95–1.03)	0.466	2,892
HDL-C	0.98	(0.90–1.06)	0.583	2,926
Creatinine	1.00	(1.00–1.00)	0.002	3,882
Sodium	0.97	(0.96–0.98)	<0.001	3,725
Potassium	0.93	(0.85–1.03)	0.172	3,736

Abbreviations: HR, hazard ratio; CI, confidence interval; CRP, C-reactive protein; Hb, hemoglobin; HbA1c, glycated hemoglobin; TG, triglycerides; LDL-C, low-density lipoprotein cholesterol; HDL-C, high-density lipoprotein cholesterol.
