# Supplementary material for: Pre-diagnostic biomarkers and risk of stress-related disorders: a cohort study based on electronic health records
Source: BMC Med. 2026 Jun 1;24:337. doi: 10.1186/s12916-026-04916-7 (PMC13227757; doi:10.1186/s12916-026-04916-7)
Supplement: Supplementary file 2 — Additional file 2 STROBE statement. [file 12916_2026_4916_MOESM2_ESM.pdf]

STROBE Statement—checklist of items that should be included in reports of observational studies

	Item No.	Recommendation	Page No.	Relevant text from manuscript
**Title and abstract**	1	(*a*) Indicate the study's design with a commonly used term in the title or the abstract	2	"We conducted a retrospective cohort study using electronic health records from Central Finland, collected between 2010 and 2023."
		(*b*) Provide in the abstract an informative and balanced summary of what was done and what was found	1–2	"We investigated whether routinely collected laboratory biomarkers are associated with stress-related disorder risk and analyzed their temporal trends before diagnosis… Hemoglobin, potassium, and LDL-C showed modest but robust associations with stress-related disorder diagnosis."
**Introduction**				
Background/rationale	2	Explain the scientific background and rationale for the investigation being reported	3–4	"Despite this theoretical understanding, empirical research on stress-related disorders remains limited and uneven across physiological systems... Whether individual routine biomarkers across different physiological systems precede stress-related disorders remains unexplored."

Objectives	3	State specific objectives, including any prespecified hypotheses	4	"Using electronic health records from Central Finland Wellbeing Services County, this study investigates whether routinely collected laboratory biomarkers are associated with future stress-related disorder diagnosis."
**Methods**				
Study design	4	Present key elements of study design early in the paper	5	"We conducted a retrospective cohort study with frequency-matched control sampling."
Setting	5	Describe the setting, locations, and relevant dates, including periods of recruitment, exposure, follow-up, and data collection	4	"The Central Finland Wellbeing Services County provides primary, secondary, and tertiary public healthcare for all residents... Our study population comprises all individuals aged 34 and above whose healthcare contact resulted in at least one laboratory measurement between January 1, 2010, and December 30, 2023."
Participants	6	(*a*) *Cohort study*—Give the eligibility criteria, and the sources and methods of selection of participants. Describe methods of follow-up<br>*Case-control study*—Give the eligibility criteria, and the sources and methods of case ascertainment and control selection. Give the rationale for the choice of cases and controls<br>*Cross-sectional study*—Give the eligibility criteria, and the sources and methods of selection of participants	4–5	"Our study population comprises all individuals aged 34 and above whose healthcare contact resulted in at least one laboratory measurement between January 1, 2010, and December 30, 2023 (n = 148,438; 52% female). … From the underlying cohort of

				148,438 individuals, all 6,758 cases diagnosed with a stress-related disorder were included. Controls (n = 67,151) were sampled from the remaining individuals to approximate the joint sex and birth year distribution of cases... To maintain comparable follow-up periods between cases and controls, each control was assigned an index date randomly drawn from case diagnosis dates within the same sex-birth-year-stratum. At baseline, participants were aged 34 to 92 years and were followed for an average of 4.6 ± 3.3 years."
		(*b*) *Cohort study*—For matched studies, give matching criteria and number of exposed and unexposed<br>*Case-control study*—For matched studies, give matching criteria and the number of controls per case	5	"Controls (n = 67,151) were sampled to approximate the joint sex and birth year distribution of cases (n = 6,758), resulting in an analytical sample of 73,909 individuals (9.1% cases)."
Variables	7	Clearly define all outcomes, exposures, predictors, potential confounders, and effect modifiers. Give diagnostic criteria, if applicable	5–6	"We defined stress-related disorders to include acute stress reaction (ICD-10 code F43.0), posttraumatic stress disorder (PTSD; F43.1), adjustment disorders (F43.2), other

				reactions to severe stress (F43.8), and unspecified reaction to severe stress (F43.9, F43). We also incorporated the diagnoses of burnout (Z73.0) and stress not elsewhere classified (Z73.3)... Chronic somatic comorbidity was quantified using the Charlson Comorbidity Index (CCI)... Prescribed medications were identified using ATC codes: antidiabetics (A10), lipid-lowering agents (C10), agents acting on the renin-angiotensin system (C09), thiazide diuretics (C03A), loop diuretics (C03C), potassium-sparing diuretics (C03D), antidepressants (N06A), and beta-blockers (C07)."
Data sources/ measurement	8*	For each variable of interest, give sources of data and details of methods of assessment (measurement). Describe comparability of assessment methods if there is more than one group	4–5	"Diagnoses and comorbidities were derived from the Finnish version of the International Classification of Diseases, Tenth Revision (ICD-10), and prescribed medications from the Anatomical Therapeutic Chemical Classification System (ATC)... All laboratory analyses were conducted according to standardized protocols of

				Central Finland Wellbeing Services County."
Bias	9	Describe any efforts to address potential sources of bias	6	"Care visit frequency was included as a proxy for informed presence bias – the tendency for individuals with more healthcare contact to have a higher probability of both laboratory testing and diagnosis."
Study size	10	Explain how the study size was arrived at	4–5	"Our study population comprises all individuals aged 34 and above whose healthcare contact resulted in at least one laboratory measurement between January 1, 2010, and December 30, 2023 (n = 148,438; 52% female). During this period, 6,758 individuals (4.6%) received a diagnosis of a stress-related disorder... Controls (n = 67,151) were sampled from the remaining individuals to approximate the joint sex and birth year distribution of cases. This resulted in an analytical sample of 73,909 individuals (9.1% cases)."

Continued on next page

Quantitative variables	11	Explain how quantitative variables were handled in the analyses. If applicable, describe which groupings were chosen and why	5–6	"The biomarkers analyzed included C-reactive protein (CRP), hemoglobin (Hb), fasting glucose, glycated hemoglobin (HbA1c), triglycerides (TG), high-density-lipoprotein cholesterol (HDL-C), low-density-lipoprotein cholesterol (LDL-C), creatinine (Cr), sodium (Na), and potassium (K), all measured from serum samples... Before statistical analysis, CRP values > 10 mg/L were excluded... Potassium values > 10 mmol/L (n = 4) were removed as biologically implausible outliers."
Statistical methods	12	(*a*) Describe all statistical methods, including those used to control for confounding	6–8	"Our analysis proceeded in two phases. First, we visualized temporal trends in biomarkers before diagnosis using generalized additive models. Second, we estimated associations between biomarker levels and stress-related disorder diagnosis within a 1-year lookback window using Cox proportional hazards models... The multivariable model additionally adjusted for CCI, care visit frequency (number of visits within the lookback period), and prescribed medications... Model assumptions were assessed using Schoenfeld residual plots for

			proportional hazards and VIFs for multicollinearity.”
	(*b*) Describe any methods used to examine subgroups and interactions	9	“We further performed exploratory subtype-specific analyses for the three largest diagnostic subgroups: acute stress reaction (n = 580), adjustment disorder (n = 538), and a combined group of other and unspecified reactions to severe stress (n = 241).”
	(*c*) Explain how missing data were addressed	8	“The model was restricted to complete cases for all exposure variables (n = 13,928; cases = 1,576). Complete-case analysis was considered appropriate because the sample size remained large, baseline biomarker values and comorbidity levels were comparable between included and excluded individuals (Additional file 1, Table S1), and potential sources of selection bias were accounted for as covariates.”
	(*d*) *Cohort study*—If applicable, explain how loss to follow-up was addressed<br>*Case-control study*—If applicable, explain how matching of cases and controls was addressed<br>*Cross-sectional study*—If applicable, describe analytical methods taking account of sampling strategy		[Not applicable: a retrospective EHR-based cohort in which all healthcare records within the study period were available for analysis, and loss to follow-up in the traditional sense does not occur.]
	(*e*) Describe any sensitivity analyses	7–9	“Robustness of these visualizations was assessed through sensitivity

analyses, restricting the cohort to individuals with at least two measurements per biomarker... To reduce the possibility that observed associations reflect pre-existing conditions, we applied a 1-year washout period, excluding cases with less than one year between their first measurement and diagnosis. To assess robustness to diagnostic heterogeneity, we first excluded individuals with Z73 codes (n = 113)... and then additionally excluded individuals with PTSD (n = 105)... We further performed exploratory subtype-specific analyses for the three largest diagnostic subgroups: acute stress reaction (n = 580), adjustment disorder (n = 538), and a combined group of other and unspecified reactions to severe stress (n = 241). Separately, to examine whether associations persisted over longer followup, we fitted univariable time-varying Cox models... As a sensitivity analysis, analogous logistic regression models were evaluated using the area under the receiver operating characteristic curve (AUC) with equivalent cross-validation."

**Results**				
Participants	13*	(a) Report numbers of individuals at each stage of study—eg numbers potentially eligible, examined for eligibility, confirmed eligible, included in the study, completing follow-up, and analysed	6–8	"The study workflow, including analytical samples at each stage of analysis, is presented in Fig. 1... From the full analytical sample, 41,514 individuals (cases = 5,046) had at least one measurement within this window... The model was restricted to complete cases for all exposure variables (n = 13,928; cases = 1,576)."
		(b) Give reasons for non-participation at each stage	4–8	"Our study population comprises all individuals aged 34 and above whose healthcare contact resulted in at least one laboratory measurement between January 1, 2010, and December 30, 2023 (n = 148,438)... From the underlying cohort of 148,438 individuals, all 6,758 cases diagnosed with a stress-related disorder were included. Controls (n = 67,151) were sampled from the remaining individuals to approximate the joint sex and birth year distribution of cases. This resulted in an analytical sample of 73,909 individuals (9.1% cases)... From the full analytical sample, 41,514 individuals (cases = 5,046) had at least one measurement within this window [and were

				included in univariable Cox models]... The [multivariable] model was restricted to complete cases for all exposure variables (n = 13,928; cases = 1,576)."
		(c) Consider use of a flow diagram	6–7	"The study workflow, including analytical samples at each stage of analysis, is presented in Fig. 1."
Descriptive data	14*	(a) Give characteristics of study participants (eg demographic, clinical, social) and information on exposures and potential confounders	9–10	"Table 1 presents baseline sample characteristics by case status... Cases were younger than controls at baseline (53.3 ± 12.2 vs. 57.8 ± 12.8 years) and more frequently female (73.6% vs. 64.4%). Comorbidity levels were low and comparable between groups."
		(b) Indicate number of participants with missing data for each variable of interest		[Sample sizes by variable are reported in Additional file 1, Table S2]
		(c) *Cohort study*—Summarise follow-up time (eg, average and total amount)	5	"At baseline, participants were aged 34 to 92 years and were followed for an average of 4.6 ± 3.3 years."
Outcome data	15*	*Cohort study*—Report numbers of outcome events or summary measures over time	9	"The analytical sample included 73,909 participants, of whom 6,758 (9.1%) were cases... Among cases, the most common first recorded diagnoses were acute stress reaction (38.6%) and adjustment disorders (33.5%) (Additional file 1, Table S3)"
		Case-control study—Report numbers in each exposure category, or summary measures of exposure		

		Cross-sectional study—Report numbers of outcome events or summary measures		
Main results	16	(*a*) Give unadjusted estimates and, if applicable, confounder-adjusted estimates and their precision (eg, 95% confidence interval). Make clear which confounders were adjusted for and why they were included	13	"In univariable models adjusted for sex and birth year [matching variables], six of ten biomarkers reached the Bonferroni-corrected significance threshold... (Table 2). In the multivariable model, additionally adjusted for CCI, care visit frequency, and prescribed medications, three biomarkers remained significant (Table 3)."
		(*b*) Report category boundaries when continuous variables were categorized		[Not applicable.]
		(*c*) If relevant, consider translating estimates of relative risk into absolute risk for a meaningful time period		[Not applicable.]

Continued on next page

Other analyses	17	Report other analyses done—eg analyses of subgroups and interactions, and sensitivity analyses	14–15	"Results in the multivariable model remained essentially unchanged when applying a 1-year washout period (Additional file 1, Table S7), when excluding Z73 diagnoses (Additional file 1, Table S8), and when additionally excluding PTSD diagnoses (Additional file 1, Table S8). In subtype-specific analyses... the direction of effects was consistent, though some associations attenuated to non-significance in the smaller subgroups (Additional file 1, Table S9). / In supplementary univariable time-varying Cox models extended over the full follow-up, only hemoglobin retained a significant association (HR 0.99 per g/L, 95% CI 0.99–0.99), while potassium and LDL-C attenuated to null (Additional file 1, Table S10)."
**Discussion**				
Key results	18	Summarise key results with reference to study objectives	15	"Three biomarkers were associated with stress-related disorder diagnosis within one year before onset... Higher potassium was associated with a reduced risk (HR 0.74 per mmol/L, 95% CI 0.64–0.86), as was higher hemoglobin (HR 0.98 per g/L, 95% CI 0.97–

				0.99). Higher LDL-C was associated with an increased risk (HR 1.12 per mmol/L, 95% CI 1.06–1.18)."
Limitations	19	Discuss limitations of the study, taking into account sources of potential bias or imprecision. Discuss both direction and magnitude of any potential bias	17	"We could not account for lifestyle and social factors – e.g., diet, body composition, smoking, alcohol use, and socioeconomic position... residual confounding from unmeasured variables remains a possibility... model discrimination was modest and evaluation was limited to a single cohort without external validation."
Interpretation	20	Give a cautious overall interpretation of results considering objectives, limitations, multiplicity of analyses, results from similar studies, and other relevant evidence	17	"While the observational design precludes causal inference, the findings may inform future causally oriented research into the somatic dimensions of stress-related pathology."
Generalisability	21	Discuss the generalisability (external validity) of the study results	18	"As with EHR research in general, our sample is conditioned on healthcare contact and having sufficient observations, which may overrepresent individuals with poorer health or higher healthcare utilization... those who rely primarily on private or occupational care – who are more likely to be higher-income and employed – may

				be slightly underrepresented in our study population.”
**Other information**				
Funding	22	Give the source of funding and the role of the funders for the present study and, if applicable, for the original study on which the present article is based	20	“A.P. was funded by the Juho Vainio Foundation. A.T. was funded by the Yrjö Jahnsson Foundation. M.V.B. and J.J. were funded by the Research Council of Finland (grants 349335 and 349336)...”

*Give information separately for cases and controls in case-control studies and, if applicable, for exposed and unexposed groups in cohort and cross-sectional studies.

Note: An Explanation and Elaboration article discusses each checklist item and gives methodological background and published examples of transparent reporting. The STROBE checklist is best used in conjunction with this article (freely available on the Web sites of PLoS Medicine at http://www.plosmedicine.org/, Annals of Internal Medicine at http://www.annals.org/, and Epidemiology at http://www.epidem.com/). Information on the STROBE Initiative is available at www.strobe-statement.org.
